# Combining UAV multisensor field phenotyping and genome-wide association studies to reveal the genetic basis of plant height in cotton (*Gossypium hirsutum*)

**DOI:** 10.1016/j.plaphe.2025.100026

**Published:** 2025-03-05

**Authors:** Liqiang Fan, Jiajie Yang, Xuwen Wang, Zhao Liu, Bowei Xu, Li Liu, Chenxu Gao, Xiantao Ai, Fuguang Li, Lei Gao, Yu Yu, Zuoren Yang

**Affiliations:** a State Key Laboratory of Cotton Bio-breeding and Integrated Utilization, Institute of Cotton Research, Chinese Academy of Agricultural Sciences, Anyang, 455000, Henan, China; b Xinjiang Key Laboratory of Crop Gene Editing and Germplasm Innovation, Institute of Western Agricultural of CAAS, Changji, 831100, Xinjiang, China; c Cotton Research Institute, Xinjiang Academy Agricultural and Reclamation Science/Northwest Inland Region Key Laboratory of Cotton Biology and Genetic Breeding (Xinjiang), Ministry of Agriculture, China; d Zhengzhou Research Base, State Key Laboratory of Cotton Bio-breeding and Integrated Utilization, School of Agricultural Sciences, Zhengzhou University, Zhengzhou, 450001, Henan, China; e College of Smart Agriculture (Research Institute), Xinjiang University, Urumqi, 830046, Xinjiang, China; f Engineering Research Centre of Cotton, Ministry of Education/College of Agriculture, Xinjiang Agricultural University, 311 Nongda East Road, Urumqi, 830052, China

**Keywords:** Plant height, UAV, RGB, LiDAR, Genome-wide association study

## Abstract

Plant height (PH) is a key agronomic trait influencing plant architecture. Suitable PH values for cotton are important for lodging resistance, high planting density, and mechanized harvesting, making it crucial to elucidate the mechanisms of the genetic regulation of PH. However, traditional field PH phenotyping largely relies on manual measurements, limiting its large-scale application. In this study, a high-throughput phenotyping platform based on UAV-mounted RGB and light detection and ranging (LiDAR) was developed to efficiently and accurately obtain time series PHs of 419 cotton accessions in the field. Different strategies were used to extract PH values from two sets of sensor data, and the extracted values were used to train using linear regression and machine learning methods to obtain PH predictions. These predictions were consistent with manual measurements of the PH for the LiDAR (R^2^ ​= ​0.934) and RGB (R^2^ ​= ​0.914) data. The predicted PH values were used for GWAS analysis, and 34 ​PH-related genes, two of which have been demonstrated to regulate PH in cotton, namely, *GhPH1* and *GhUBP15*, were identified. We further identified significant differences in the expression of a new gene named *GhPH_UAV1* in the stems of the *G. hirsutum* cultivar ZM24 harvested on the 15th, 35th, and 70th days after sowing compared with those from a dwarf mutant (*pag1*), which presented shortened stem and internode phenotypes. The overexpression of *GhPH_UAV1* significantly promoted cotton stem development, whereas its knockout by CRISPR-Cas9 dramatically inhibited stem growth, suggesting that *GhPH_UAV1* plays a positive regulatory role in cotton PH. This field-scale high-throughput phenotype monitoring platform significantly improves the ability to obtain high-quality phenotypic data from large populations, which helps overcome the imbalance between massive genotypic data and the shortage of field phenotypic data and facilitates the integration of genotype and phenotype research for crop improvement.

## 1 Introduction

Cotton (*Gossypium* spp.) is an important commercial crop and a major producer of natural fiber for the global textile industry [1]. Upland cotton (*Gossypium hirsutum* L.), the most widely cultivated cotton, accounts for more than 90 ​% of the global cotton yield [2]. Cotton architecture, as a comprehensive agronomic trait, is defined as the three-dimensional structure of the aerial parts [3]. The core of plant architecture is shaped by plant height, stem growth habit, and branching pattern [4]. Cotton architecture is a key factor influencing the yield and mechanized cultivation efficiency of cotton. Improving cotton plant architecture can accelerate the breeding process and improve cotton yield and quality, thus increasing the economic benefits of cotton [5]. The main cultivation characteristics of cotton in the Xinjiang Uygur Autonomous Region of China are dwarfism, density, and earliness, with dwarfism referring to plant height, which contributes significantly to cotton production.

Plant height (PH), an important trait in cotton architecture, can be optimized to increase planting density, thereby improving the efficiency and yield of mechanized cotton harvesting [6,7]. During the Green Revolution of the 1960s, scientists altered plant architecture by introducing dwarf genes into crops, significantly increasing yields [[8], [9], [10]]. However, improving PH through traditional breeding methods requires considerable time in the field and may be less efficient. The genomics revolution has improved the efficiency of crop breeding. Identifying genes related to PH via genome-wide association studies (GWASs) and exploring the genetic mechanism of PH are current research hotspots. Su et al. identified the *Gh_D03g0922* gene, which controls PH, through a GWAS of 355 upland cotton accessions [11]. Wen et al. recognized five QTLs affecting PH through GWAS using 121 cotton accessions [12]. Wang et al. identified 68 gene loci related to PH through a restricted two-stage multilocus GWAS using 315 cotton accessions [13]. Because PH changes rapidly during cotton growth periods, it is critical to obtain rapid PH phenotypic traits of large germplasm populations for GWAS. However, phenotypic changes throughout the cotton life cycle cannot be comprehensively tracked by manual measurements, and high-throughput PH phenotyping in the field requires continuous research.

Traditional field PH value acquisition relies on manual measurements, which have significant drawbacks such as low throughput, high cost, and subjectivity. Some studies have improved throughput to some extent by obtaining phenotyping data through indoor phenotyping platforms [14], but the high costs of these facilities and the inability to mimic field growth environments greatly limit their application. The rapid advances in unmanned aerial vehicle (UAV) technology and imaging analysis over the past decade have opened new avenues for high-throughput monitoring of crop field phenotypes [15,16], facilitating the availability of accurate phenotypic data for GWAS analysis [17]. High-throughput phenotyping allows dynamic observations of multiple traits in large populations at different growth stages and provides more objective trait characterization from images or spectra than traditional methods [18]. The main methods of extracting the PH using UAV remote sensing technology include constructing a point cloud model using UAV-mounted LiDAR to extract the PH and using a digital camera to collect surface models of the experimental area and then subtracting these models. These methods have been applied to many crops, such as cotton [[19], [20], [21]], wheat [[22], [23], [24]], maize [25,26], and rice [[27], [28], [29]]. Encouragingly, Ye et al. successfully identified genes significantly associated with PH by acquiring field cotton PH data from UAV RGB images, analyzing them by linear regression and then combining them with genotypic populations for GWAS analysis [30]. However, the feasibility of using UAV multisensor data after linear regression and machine learning analysis to assess the efficiency of time series PH monitoring in large fields combined with GWASs to identify genes regulating plant height requires urgent investigation.

In this study, we used a UAV equipped with RGB and LiDAR sensors to acquire images of 419 cotton accessions at five time points before manual topping in the Xinjiang Uygur Autonomous Region. We employed different strategies for PH extraction from two types of sensor data and trained the extracted values using linear regression and machine learning methods to obtain PH predictions. PH predictions were used for GWAS, which not only identified two genes that have been shown to regulate cotton PH but also identified a new gene through a short-stem mutation and experimentally verified that it positively regulates cotton PH.

## 2 Materials and methods

### 2.1 Field experimental design

**Fig. 1:**
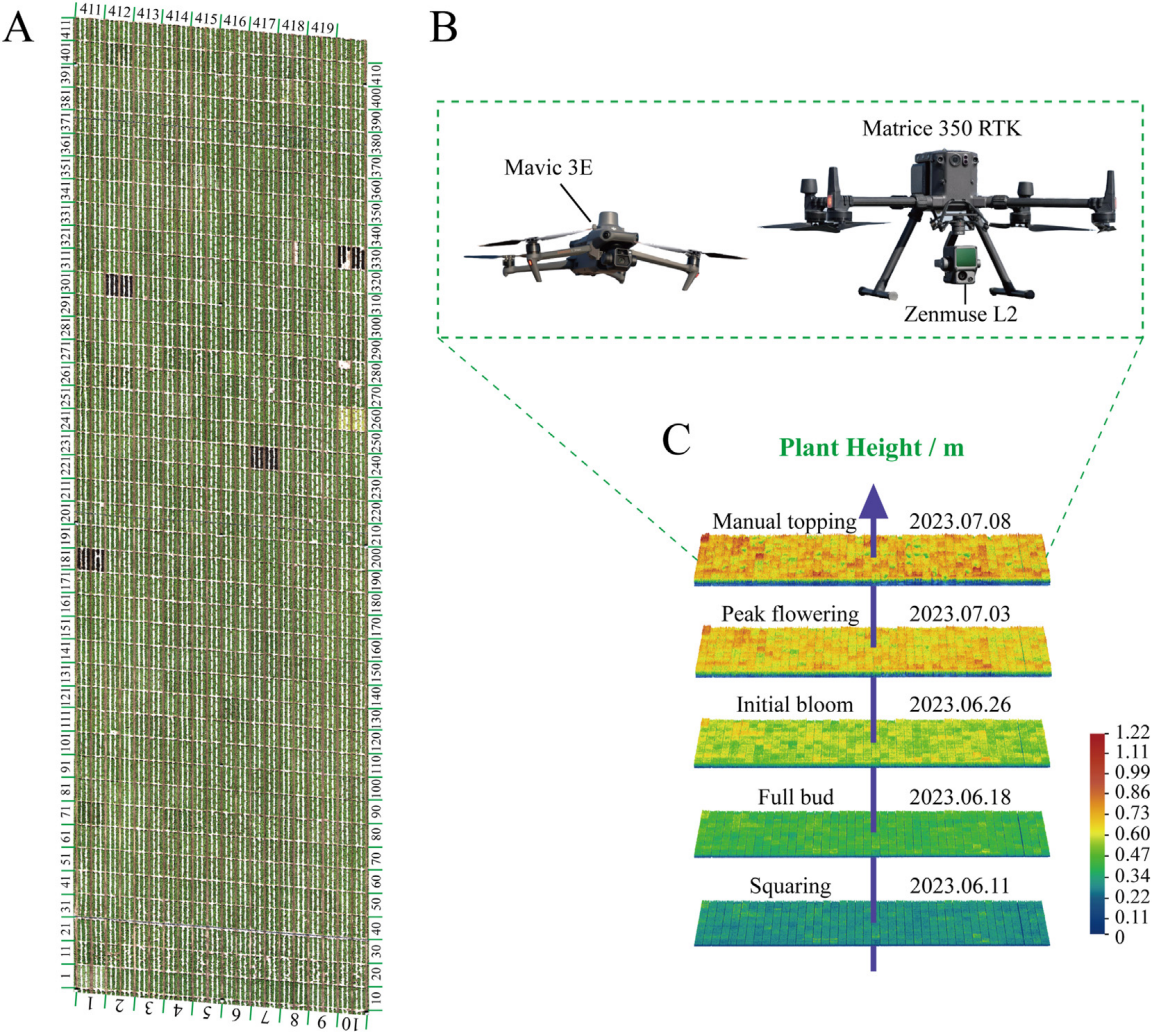
**Orthophotographs and UAV image acquisition of the study area.** (A) Orthophotos of 419 upland cotton plots at the experimental site. (B) UAV systems and integrated sensors. (C) Scheme for plant height UAV image acquisition at five time points in cotton.

A total of 419 upland cotton (*G. hirsutum* L.) accessions were used in this study [2], and specific information is provided in Table S1. A field experiment was conducted in 2023 in Shihezi city, Xinjiang, China (85.99′E, 44.31′N). The field was divided into 419 plots, consisting of one row and six columns, each with the same dimensions of 4.5 ​m ​× ​5 ​m. Each accession was planted in a single plot (Fig. 1A). Cotton seeds were sown on May 15, 2023, and the manual topping was performed on September 8, 2023.

### 2.2 UAV image acquisition and experimental field measurement

The UAV platforms used for image acquisition in the experimental field were all DJI products (Fig. 1B). Specifically, cotton phenotypic information was collected using RGB sensors mounted on the DJI Mavic 3E and a LiDAR sensor on the DJI Matrice 350 RTK. The DJI Mavic 3E has a mechanical shutter wide-angle camera and a 4/3 CMOS sensor with 20 million effective pixels. The DJI Matrice 350 RTK (M350) was equipped with a high-precision mapping LiDAR sensor (Zenmuse L2), which integrated a frame LiDAR and a high-precision self-developed inertial guidance system. Both UAVs mentioned above were equipped with an advanced integrated real-time kinematic (RTK) GNSS module that can provide positioning data.

These flights were carried out from June 11, 2023 (squaring stage), to July 8, 2023 (manual tipping), during which the cotton plants grew most rapidly (Fig. 1C). The UAVs were flown from 12:00 p.m. to 4:00 p.m. under clear skies and low-wind conditions (Table 1). The ground-truth PH was measured manually from the ground to the top of the stem on UAV flight days. Twenty-four sample plots were randomly selected at time 1, and the PH values of the same plots were measured at the following four time points (time 2–5) to create the training dataset. The actual PH data for all 419 plots were measured at time 4 and time 5 for the final testing dataset. For each cotton population plot, the PH of 12 randomly selected plants was measured and averaged.

**Table 1:** Detailed flight parameters of UAVs.

Parameters	DJI Mavic 3E	DJI Matrice 350 RTK
Relative flight altitude	15m	20m
Flight speed	3.3m⋅s^−1^	2.2m⋅s^−1^
Side overlap rate	70 ​%	70 ​%
Forward overlap rate	80 ​%	80 ​%
Flight period	19min 1s	15min 32s
Ground sample distance	0.4 ​cm⋅px^−1^	0.54 ​cm⋅px^−1^
Controlled rotation range	Tilt: −90° to +35°	Tilt: −120° to +30°
Main line angle	352°	176°

### 2.3 Methods for RGB and LiDAR data processing

**Fig. 2:**
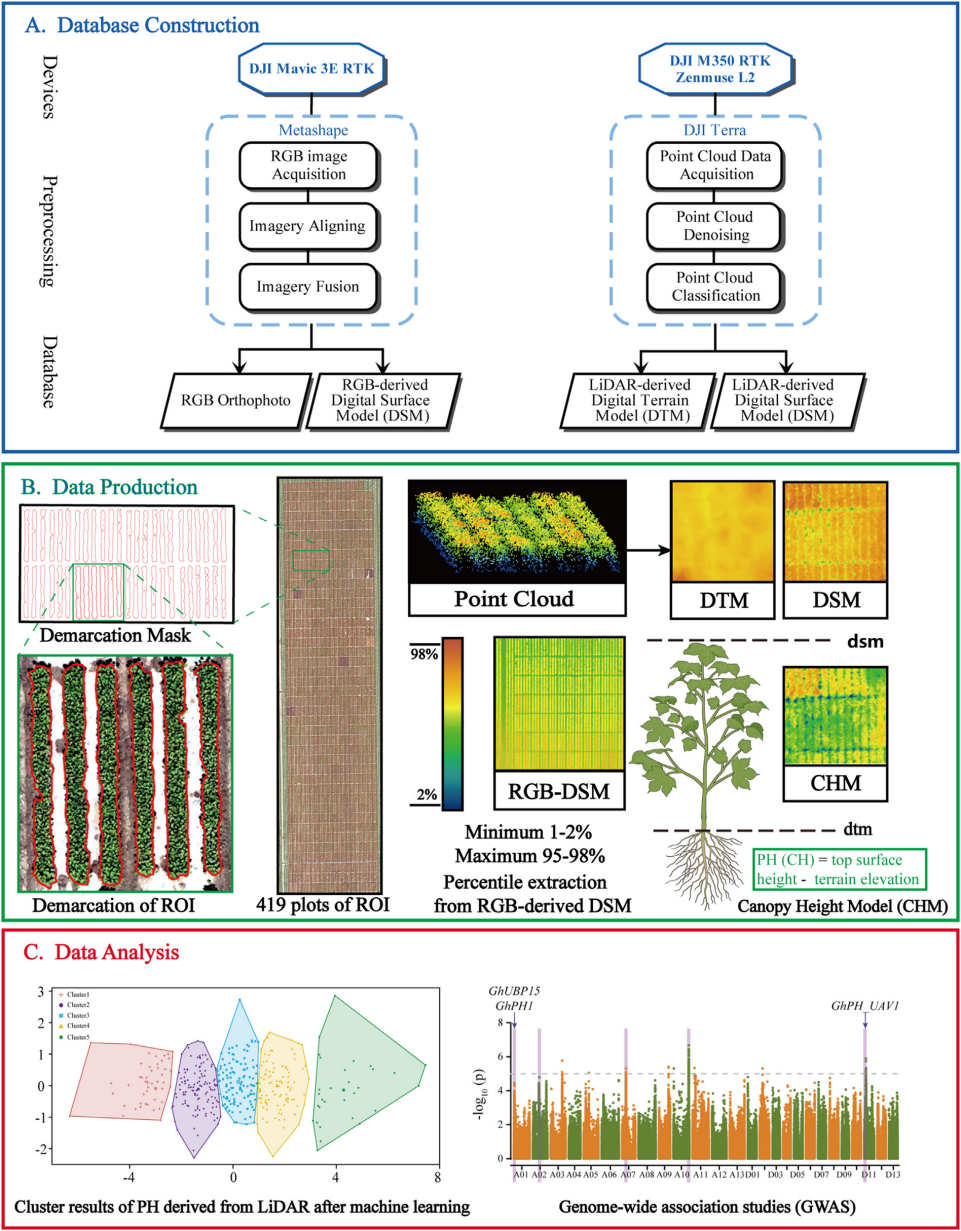
**Flow chart of this study.** (A) Processing steps for RGB and LiDAR data. (B) Each of the 419 cotton plots was precisely demarcated. The difference between the first and 95th percentiles was used to extract the PH from the on-season digital surface model (DSM) of the plot RGB images. The PH was calculated as the difference between the DSM and digital terrain model (DTM) of the plot LiDAR data. (C) UAV-based PH was used for K-means clustering and a genome-wide association study (GWAS).

The data processing program was divided into three main steps: data construction (Fig. 2A), data production (Fig. 2B), and data analysis (Fig. 2C). Precise demarcation of the cotton population minimizes the influence of the soil. For each of the 419 cotton accessions, a demarcation mask was created. The field was demarcated five times to match the phenotypic changes in the cotton, such as the increasing canopy area, at the five time points. When determining the range, it was essential to ensure that the demarcation accurately encompassed the cotton canopy. The processing of data from each of the two sensor types (RGB and LiDAR sensors) is described below.

After raw RGB photos were acquired from the Mavic 3E wide-angle camera, preprocessing was needed. Data processing was performed using Agisoft Metashape V2.1.0. RGB preprocessing included image alignment, camera alignment optimization, and the construction of dense point clouds, depth maps, digital surface models (DSMs), and digital orthophoto models (DOMs). Image alignment was conducted via UAV aerial triangulation. Subsequently, depth maps were generated via the image alignment process, and further point clouds and DSMs were generated. PH extraction was performed using the percentile extraction method. Considering that soil conditions are influenced by factors such as human activities and rainfall, images of bare ground before sowing were not taken in this study; rather, the difference between the upper and lower percentiles of the DSM was used as the UAV-predicted PH values. The minimum value of the 1%–2% percentiles was chosen as the ground-truth PH. The maximum value of the 95%–98 ​% percentiles was chosen as the height at the top of the stem [30].

To obtain a real cotton point cloud, the data acquired by UAV-LiDAR had to be preprocessed. This involved data interpretation, point cloud resampling, and denoising. Data interpretation was performed using DJI Terra V4.2.2 and included point cloud alignment, precision optimization, ground point cloud classification, and digital terrain model (DTM) building. After interpretation, the point cloud data were saved in LAS format. To maintain the independence and clarity of the point cloud, resampling was necessary. Additionally, denoising was performed to reduce noise in the point cloud. Based on the LAS file, the DSM was built using the structure-from-motion (SfM) technique. The resolutions of the DSM and DTM derived from UAV-LiDAR were as high as 0.01 ​m. DTM generation was based on the classification result of ground point clouds without plants. The DSM provides the top height of the cotton canopy, and the DTM provides the elevation information of the same plant. Therefore, plant height (PH) was calculated as the difference between the DSM and DTM, as shown in the following formula: Canopy Height Model (CHM) ​= ​DSM – DTM.

### 2.4 Linear regression, machine learning and model validation

In this study, linear regression (LR) models were established to determine the relationship between the UAV-based PH and the manually measured PH values. Furthermore, 12 machine learning regression algorithms were used: Random forest regression (RFR), K-nearest neighbor regression (KNN), Huber regression (Huber), Least angle regression (LAR), Ridge regression (RR), Light gradient boosting machine (LightGBM), Decision tree regression (DTR), Extra trees regression (ETR), Orthogonal matching pursuit (OMP), Bayesian ridge (BR), AdaBoost regression (AdaBoost), and CatBoost regression (CatBoost). All algorithms were implemented in MATLAB R2023b. Although many regression methods exist for estimation, different machine learning methods have varying effects. In this study, the most applicable machine learning models for each of the two sensors (RGB and LiDAR sensors) were determined through computational and accuracy screening. The cotton PH was measured via RGB-DSM, and the LiDAR point cloud was verified against the manually measured PH. The extraction accuracy of plant height was evaluated using four indicators: the coefficient of determination (R^2^), mean absolute error (MAE), mean square error (MSE), and root mean square error (RMSE). The larger R^2^ value and the smaller MAE, MSE, and RMSE values suggest that the model is more accurate and precise in estimating the PH value. The formulas for R^2^, MAE, MSE, and RMSE are as follows:

$$R^{2}=1-\frac{\sum_{i=1}^{n}{\left(Z_{mi}-Z_{pi}\right)}^{2}}{\sum_{i=1}^{n}{\left(Z_{mi}-\bar{Z_{m}}\right)}^{2}}$$

$$\text{MAE}=\frac{1}{n}\sum_{i=1}^{n}\left|Z_{mi}-Z_{pi}\right|$$

$$\text{MSE}=\frac{1}{n}\sum_{i=1}^{n}{\left(Z_{mi}-Z_{pi}\right)}^{2}$$

$$\text{RMSE}=\sqrt{\frac{1}{n}\sum_{i=1}^{n}{\left(Z_{mi}-Z_{pi}\right)}^{2}}$$

Z_pi_ and Z_mi_ are the predicted and measured plant heights, respectively, n is the number of height values, and $\bar{Z_{m}}$ is the average measured PH.

### 2.5 Classification of cotton accessions based on PH value dynamics

To compare the PH values of the 419 cotton accessions at the five time points, K-means clustering [31] was conducted to classify the cotton accessions into different clusters. The sums of squares for different k values were compared, and the optimal number of clusters for which the slope does not change significantly was selected. After confirming the best clusters, the dynamic changes in the plant height of the samples in each cluster at the five time points were visualized using the software MeV v4.9 [32].

### 2.6 Genome-wide association studies for the PH trait

Genome resequencing and assessment of genomic variation in these 419 upland cotton accessions generated 3,665,030 SNPs, as described previously [2]. In this study, SNPs were filtered using PLINK [33], and 1,980,539 high-quality SNPs (MAF >0.05, missing rate <10 ​%) were identified and analyzed by GWAS through the software EMMAX. The best linear unbiased estimate (BLUE) was calculated using PH values from three replicate plots for each of the 419 cotton accessions and then used in the GWAS. The significance threshold of association was set as −log10 (p) ​= ​5 (control threshold). The Manhattan plot of the GWAS results was generated using the “CMPlot” package.

### 2.7 RNA library construction and sequencing

We previously constructed a *pagoda1* (*pag1*) mutant, which exhibits a variety of phenotypic traits, the most obvious being dwarfism, with shortened stems and internodes [34]. Stem samples of *pag1* and the *G. hirsutum* cultivar ZM24 were harvested on the 15th, 35th, and 70th days after sowing for transcriptome sequencing.

RNA from stem tissues was extracted using an RNA Purification Kit, after which RNA-seq libraries were produced. After library construction, RNA sequencing was conducted with a sequencing read length of PE150. Clean reads were then mapped to the Zhongmiansuo 24 (ZM24) genome using HISAT2, and gene expression levels in ZM24 and *pag1* stem tissues were calculated.

### 2.8 Vector construction and cotton transformation

The coding sequence of *GhPH_UAV1* (*Ghicr24_D11G117900*) was extracted from the CottonGen database. The *GhPH_UAV1* gene was amplified from the cDNA library of ZM24 using primers (Table S2) and subsequently inserted into the 35S promoter-driven pCAMBIA-2300 vector to construct the *GhPH_UAV1* overexpression vector. To construct the *GhPH_UAV1* CRISPR/Cas9 knockout vector, we designed sgRNAs for *GhPH_UAV1* using the software CRISPR-P. The sgRNA sequence of *GhPH_UAV1* was subsequently inserted into the CRISPR/Cas9 knockout vector.

After sterilization, ZM24 seeds were incubated at 30 ​°C in a light-deficient room for seven days. All the vectors used for *GhPH_UAV1* overexpression and *GhPH_UAV1* CRISPR/Cas9 were separately incorporated into the *Agrobacterium* strain LBA4404. Hypocotyl segments were cut into 5-mm segments as explants and then immersed in *A*. *tumefaciens* suspension for 15 ​min. After callus induction, proliferation, embryogenic callus induction, embryo differentiation, and plantlet regeneration, the putative transgenic cotton plants were transferred to pots and placed in a greenhouse for cultivation [35]. The expression levels of *GhPH_UAV1* in the overexpressing plants (*GhPH_UAV1*-OE) were determined via qRT‒PCR. CRISPR/Cas9 knockout cotton plants (*GhPH_UAV1*-Cas9) were identified through high-throughput sequencing analysis.

### 2.9 Quantitative RT‒PCR analysis

The qRT‒PCR program was as follows: 5 ​min of initial denaturation followed by 40 cycles of 15 ​s of denaturation (95 ​°C), 15 ​s of annealing (60 ​°C), and 20 ​s of extension (72 ​°C). The *GhHistone3* gene (GenBank accession no. AF024716) was employed as an internal control. Relative changes in gene expression were estimated via the 2^−ΔΔCT^ method [36].

## 3 Results

### 3.1 Estimation of PH via RGB and LiDAR using linear regression

**Fig. 3:**
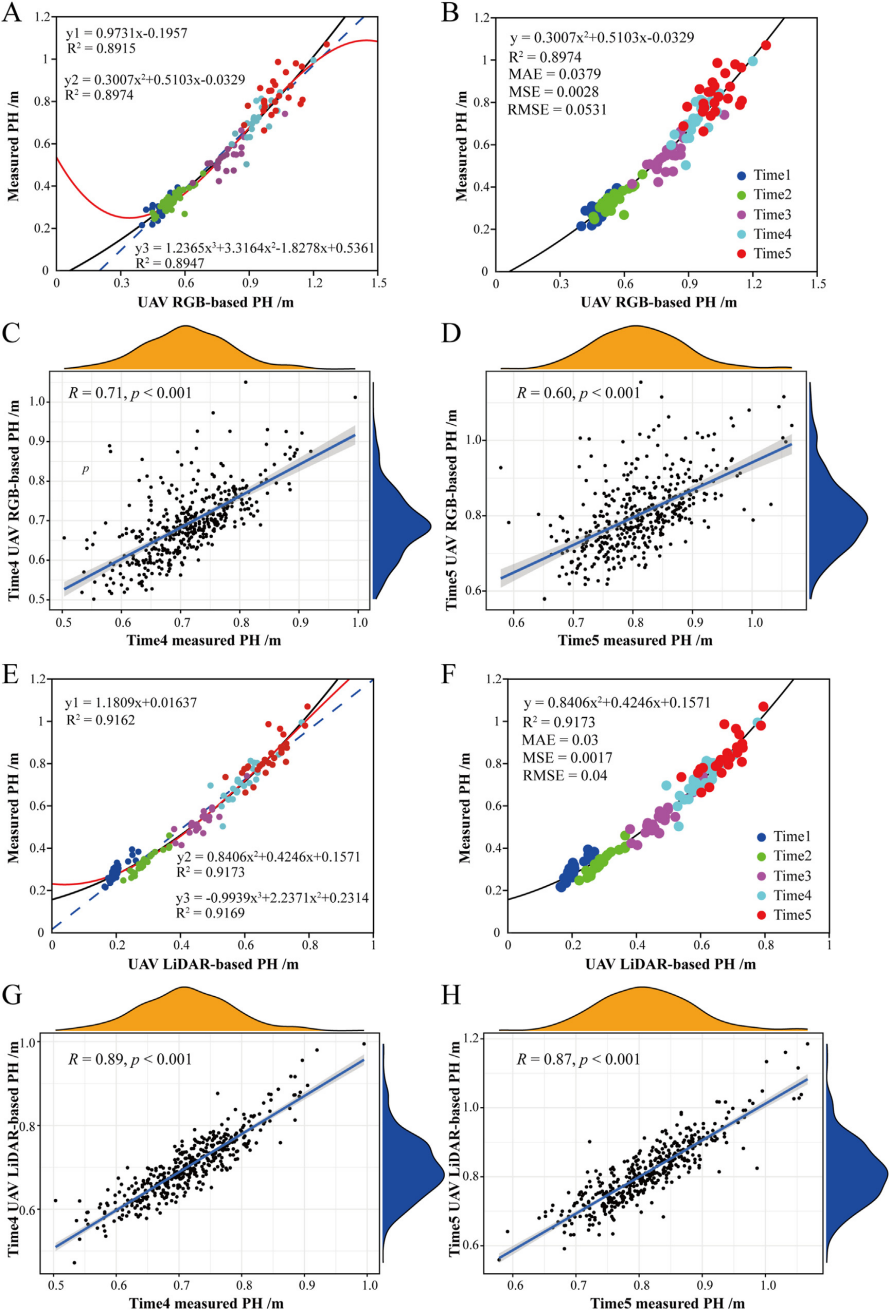
**Prediction of PH values extracted from the RGB and LiDAR data using linear regression and correlation analysis.** Three fitted equations in the linear regression analysis of the relationship between RGB-based PH values (A)/LiDAR-based PH values (E) and manually measured PH values. Second-degree polynomial regression had the best fit between the RGB-based PH values (B)/LiDAR-based PH values (F) and the manually measured PH values. Correlation between RGB-based PH predictions (C–D)/LiDAR-based PH predictions (G–H) and manually measured PH values of 419 cotton accessions at Times 4 and 5 after linear fitting.

We phenotyped 419 cotton accessions at five time points using UAV-mounted RGB and LiDAR (Fig. 1C and D) and extracted PH values from the UAV images (Fig. 2A; Table S3). Linear regression was first employed to analyze the relationship between the UAV-based PH and the manually measured PH. We manually measured 24 sample plots with a ruler at all five time points (Table S4) and derived the corresponding predicted values of PH through linear regression. The results of the three fitted equations from the linear regression analysis showed that the second-degree polynomial regression had the best fit, with a coefficient of determination (R^2^) of 0.8974 between the RGB-based extracted PH and the manually measured PH (Fig. 3A). The mean absolute error (MAE) was 0.0379, the mean square error (MSE) value was 0.0028, and the root-mean-square error (RMSE) value was 0.0531. To evaluate the accuracy of the predicted PH values, we measured all 419 sample plots at time points 4 and 5 and performed Spearman correlation analysis. The results revealed that the correlations between the predicted PH values extracted from the RGB images after linear fitting and the manually measured PH values were 0.71 (time 4, Figs. 3C) and 0.60 (time 5, Fig. 3D), respectively.

Similarly, the results of the three fitted equations from the linear regression analysis showed that the second-degree polynomial regression had the best fit, with a coefficient of determination (R^2^) of 0.9173 between the LiDAR-based extracted PH values and the manually measured PH values (Fig. 3E). The MAE was 0.03, the MSE was 0.0017, and the RMSE was 0.04 (Fig. 3F). Fig. 3G–H shows good correlations between the predicted PH values extracted from the LiDAR data after linear fitting and the manually measured PH values, with R ​= ​0.89 ​at time 4 (Fig. 3G) and R ​= ​0.87 ​at time 5 (Fig. 3H).

### 3.2 Estimation of PH based on the RGB and LiDAR data using machine learning methods

**Fig. 4:**
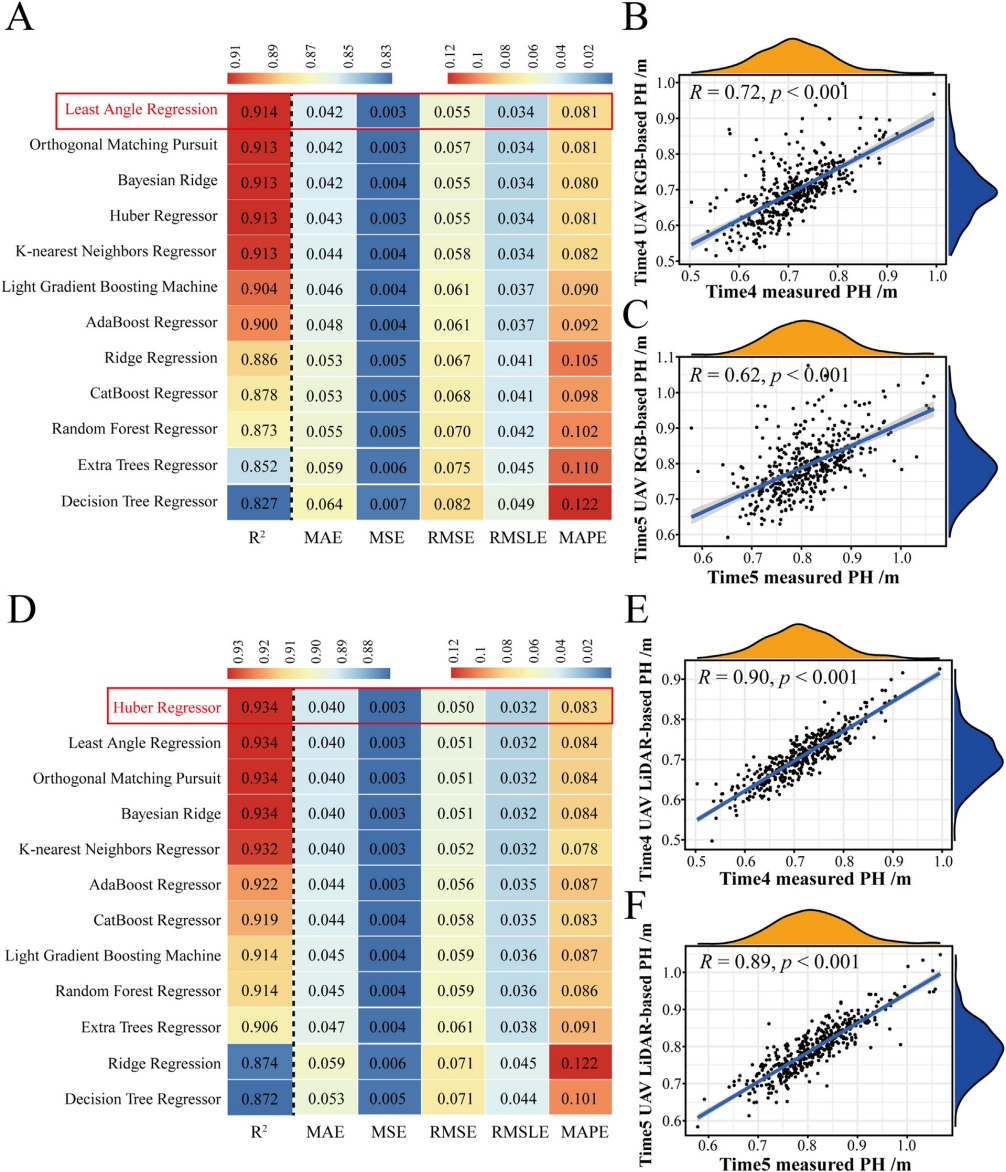
**Prediction of PH values extracted from RGB and LiDAR data using machine learning methods and correlation analysis.** (A) Estimation of PH via RGB (A)/LiDAR (D) using 12 machine learning methods. Least angle regression and Huber regression may have greater potential for predicting the PH values of the 419 cotton accessions at different growth stages. Correlation between RGB-based PH predictions (B–C)/LiDAR-based PH predictions (E–F) and manually measured PH values of 419 cotton accessions at Times 4 and 5 after least angle regression and Huber regression analysis, respectively.

To compare prediction accuracy, the PH values extracted from the RGB images at five time points were also independently predicted by 12 machine learning models, and the evaluation results are shown in Fig. 4A. The least angle regression model had the largest R^2^ (0.914) and the smallest MAE (0.042), MSE (0.003), RMSE (0.055), RMSLE (0.034), and MAPE (0.081) relative to the other 11 machine learning models. The correlations between the PH values extracted from the RGB images after prediction by least angle regression and the manually measured PH values were 0.72 (Time 4, Figs. 4B) and 0.62 (Time 5, Fig. 4C), respectively.

Similarly, the Huber regressor had the largest R^2^ (0.934) and the smallest MAE (0.040), MSE (0.003), RMSE (0.050), RMSLE (0.032), and MAPE (0.083) values relative to those of the other 11 machine learning models (Fig. 4D). The correlations between the PH values extracted from the LiDAR data after prediction by the Huber regressor and the manually measured PH values were 0.90 (Time 4, Figs. 4E) and 0.89 (Time 5, Fig. 4F), respectively. Thus, least angle regression and Huber regression may have greater potential for predicting the PH values of the 419 cotton accessions at different growth stages.

### 3.3 Clustering analysis of the 419 cotton accessions

**Fig. 5:**
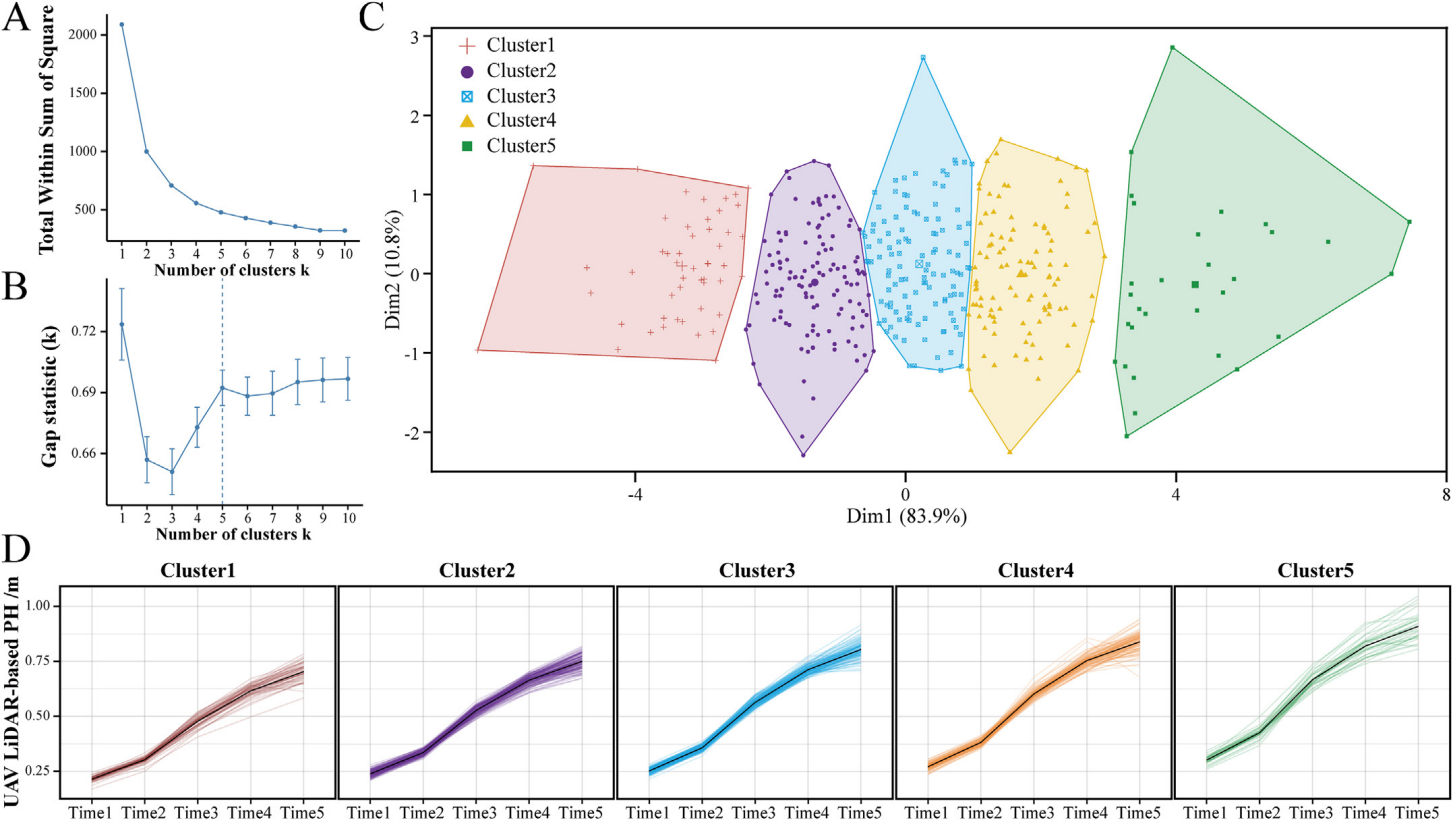
**Clustering analysis and PH dynamics of the 419 cotton accessions at different growth stages.** (A) The sum of squares for different k values. The best cluster number was chosen where the slope change was not obvious (k ​= ​5). (B) Gap statistics for different k values. The best cluster number was chosen where the gap value was the largest (k ​= ​5). (C) K-means clustering results of the 419 cotton accessions, with different colors representing different clusters. (D) Time-dependent cotton PH dynamics among the five clusters based on K-means clustering.

After comparing the PH values obtained from the above UAV data, we chose the predicted PH obtained from LiDAR through the Huber regressor model for K-means cluster analysis to examine the similarity of the 419 cotton accessions over time. The optimal number of clusters was determined to be five (Fig. 5A–B), namely, Cluster 1, Cluster 2, Cluster 3, Cluster 4, and Cluster 5. Polygons of different colors represent the different clusters. There was no obvious overlap of the polygons, indicating clear distinctions between the five clusters (Fig. 5C). Clusters 1, 2, 3, 4, and 5 contained 122, 102, 30, 49, and 116 cotton accessions, respectively (Table S5).

Based on the results of the clustering analysis, the differences in the dynamic changes in PH were analyzed. Fig. 5D shows the changes in the five clusters, such as the maximum PH value and the rate of ascent, at the five time points. The curves indicate that the PH value tended to increase and then peaked. A sequential rate of increase in Clusters 1–5 was observed, along with the final maximum PH value reached, with Cluster 5 reaching a maximum PH value at Time 5.

### 3.4 GWAS analysis for PH at the five time points based on the LiDAR- and RGB-based PH predictions

**Fig. 6:**
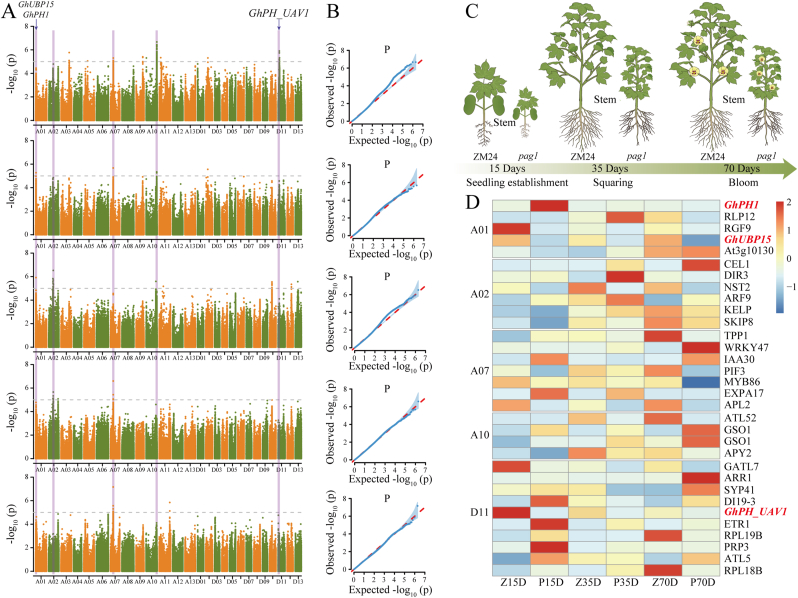
**Manhattan plots and QQ plots for plant height (PH) and transcriptome results at five stages.** Manhattan plots (A) and QQ plots (B) of genome-wide association study (GWAS) results based on the LiDAR-based PH prediction values with the highest confidence after least angle regression analysis. The dashed line corresponds to the Bonferroni-corrected thresholds of p ​= ​1.00 ​× ​10^−5^ (−log_10_ p ​= ​5). The arrowheads indicate significant PH-associated quantitative trait loci. (C) Schematic representation of the design for the stem tissues. (D) Heatmap showing the expression patterns of the *GhPH_UAV1* gene in cotton stems at five stages in *pag1* and its wild-type counterpart Zhongmiansuo 24 (ZM24). The 15th-day stems of ZM24 (Z15D) or *pag1* (P15D); the 35th-day stems of ZM24 (Z35D) or *pag1* (P35D); and the 70th-day stems of ZM24 (Z70D) or *pag1* (P70D).

**Fig. S1:**
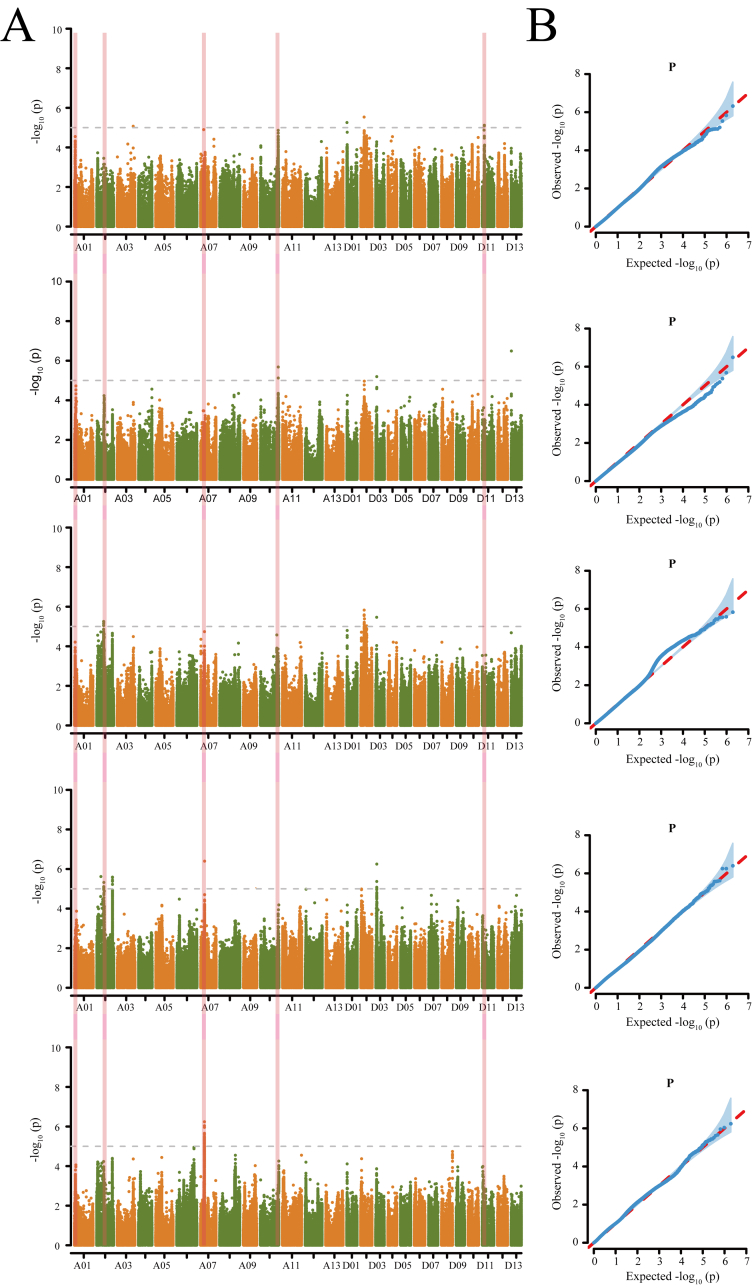
Manhattan plots and QQ plots for plant height (PH) at five time points. Manhattan plots (A) and QQ plots (B) of GWAS results based on the RGB-based PH prediction values with the highest confidence after least angle regression analysis. The dashed line corresponds to the Bonferroni-corrected thresholds of p ​= ​1.00 ​× ​10^−5^ (−log_10_ p ​= ​5). The arrowheads indicate significant PH-associated quantitative trait loci.

GWAS was carried out using the BLUE values for PH at the five time points for the 419 cotton accessions. In total, 1465 SNPs were notably correlated with PH according to the LiDAR-based PH prediction values at the five time points. We then detected five peaks on chromosomes A01 (∼3.75–∼3.88 ​Mb), A02 (∼43.8–∼44.5 ​Mb), A07 (∼27.8–∼28.2 ​Mb), A10 (∼110.7–∼111.1 ​Mb), and D11 (∼10.8–∼11.0 ​Mb) that were significantly associated with PH and overlapped at more than one time point (Fig. 6A–B; Table S6). The RGB-based PH prediction values were also analyzed by GWAS with genotypic data, and the same five significant signals were identified to exist simultaneously at multiple time points (Fig. S1). There were 90 genes within the above significant interval, of which only 34 had annotation information (Table S7). Notably, there were five genes in this 0.13 ​Mb region on chromosome A01, which is consistent with the region localized by cotton plant height measurement using UAV remote sensing phenotyping platforms (*GhUBP15*) [30] and manual measurement (*GhPH1*) [37] of cotton PH (Table S7). Compared with the *G. hirsutum* cultivar ZM24, the *pag1* mutant presented shortened stems and internodes (Fig. 6C). Analysis of the stem transcriptome data revealed that *GhPH1* gene expression was significantly greater in the 15th-day stems of *pag1* than in those of ZM24 and that the *GhPH1* gene negatively regulated cotton PH; *GhUBP15* gene expression in *pag1* was significantly lower than that in ZM24 in the 15-, 35-, and 70-day stems, and the *GhUBP15* gene positively regulated cotton PH (Fig. 6D). These findings are consistent with published studies and demonstrate the reliability of the transcriptomic data. Additionally, we identified a new gene, *GhPH_UAV1*, with an expression trend similar to that of *GhUBP15* and hypothesized that this gene might regulate plant height variation.

### 3.5 GhPH_UAV1 positively regulates cotton PH

**Fig. 7:**
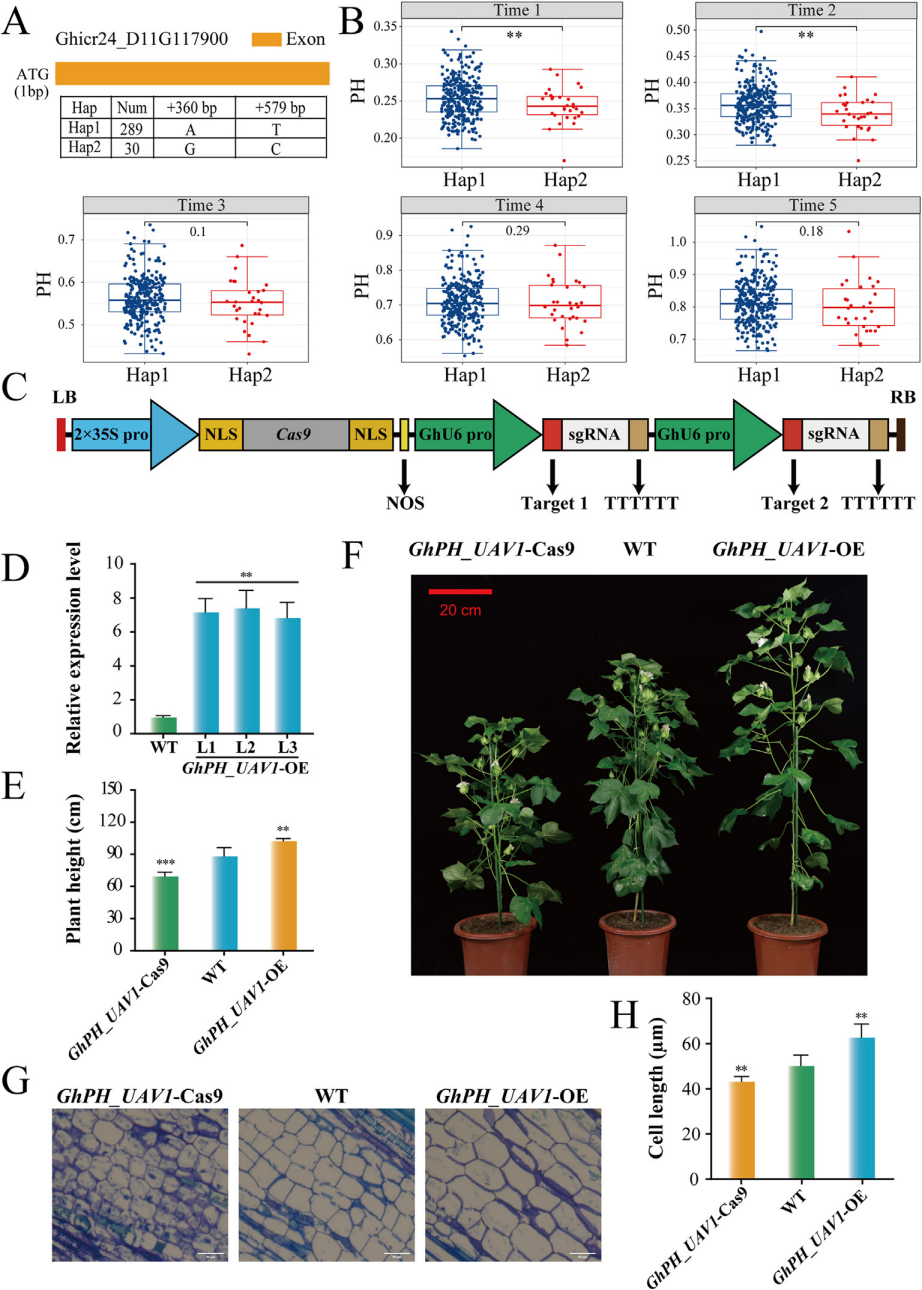
The *GhPH_UAV1* gene positively regulates cotton stem development. (A) Gene structure of Ghicr24_D11G117900 (*GhPH_UAV1*). Two haplotypes observed in the cotton accessions are listed. (B) Box plot of PH at different time points, based on the two haplotypes of two SNPs in *GhPH_UAV1* (∗∗*P* ​< ​0.01, by analysis of variance). (C) Schematic diagram of CRISPR/Cas9 vector construction. (D) *GhPH_UAV1* expression in the stem tissues of the *GhPH_UAV1*-OE (overexpression) lines. (E) Stem height of *GhPH_UAV1*-OE/Cas9 and its wild-type (WT) counterpart Zhongmiansuo 24 (ZM24). The error bars represent the SDs for 30 different stems of *GhPH_UAV1*-OE/Cas9 and ZM24. (F) Phenotypes of *GhPH_UAV1*-OE/Cas9 and ZM24 stems. Bar ​= ​20 ​cm. (G) Stem cell length of the *GhPH_UAV1*-OE/Cas9 plants. Scale bar ​= ​50 ​μm. (H) Statistics of cell lengths. The data presented are from three independent experiments. The results are expressed as the means ​± ​SDs of triplicate experiments. Statistical significance was determined using one-way ANOVA combined with a *t*-test (∗∗*P* ​< ​0.01, ∗∗*P* ​< ​0.001).

**Fig. S2:**
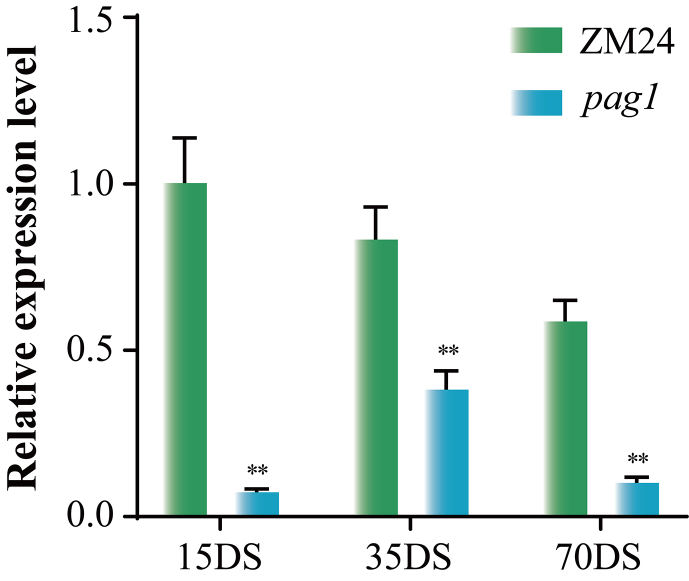
Expression levels of the *GhPH_UAV1* gene in stem 15, stem 35, and stem 70 of ZM24 and *pag1*.

**Fig. S3:**
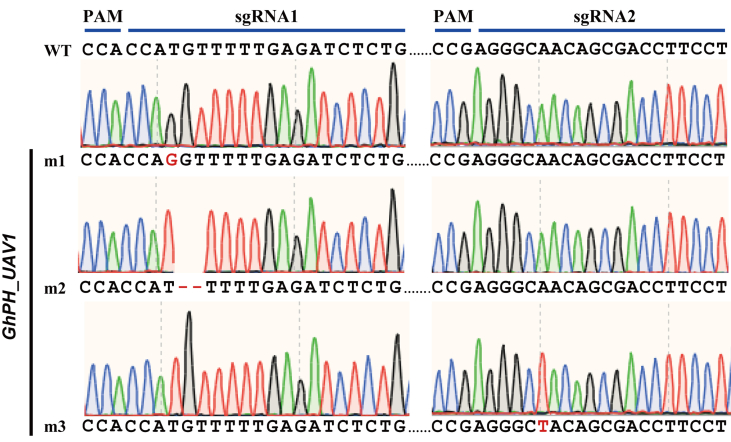
The detection of mutation types in the *GhPH_UAV1* strain via PCR sequencing revealed alterations in the *GhPH_UAV1* gene. The locations where sgRNA1 and sgRNA2 target the *GhPH_UAV1* gene are depicted. Red horizontal lines represent deletions in the corresponding bases, whereas replaced nucleotides are highlighted in red. The mutations observed, denoted as m1-m3, correspond to three distinct *GhPH_UAV1* knockout strains.

**Fig. S4:**
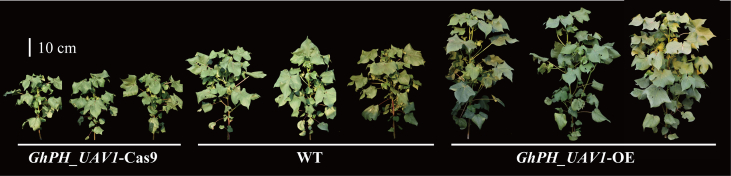
Phenotypes of the stems of *GhPH_UAV1*-OE/Cas9 and the wild-type (WT) counterpart Zhongmiansuo 24 (ZM24) under laboratory growth conditions. Bar ​= ​10 ​cm.

*GhPH_UAV1* expression was notably decreased in the stems of *pag1* (P15D, P35D, and P70D) compared with those of ZM24 (Z15D, Z35D, and Z70D) at 15, 35, and 70 days (Fig. 6D). Compared with those of ZM24, the expression of *GhPH_UAV1* at 15DS, 35DS and 70DS was downregulated by 92.27 ​%, 53.96 ​%, and 82.03 ​%, respectively, in *pag1* stems (Fig. S2). Further screening revealed two nonsynonymous SNPs: D11_10957680 and D11_10957899. These SNPs formed two haplotypes (Fig. 7A). The accessions with the alternative GC sequence (Hap2) showed significantly shorter PHs than those with the AT sequence (Hap1) (Fig. 7B). To further elucidate the role of *GhPH_UAV1* in cotton PH, we generated *GhPH_UAV1* knockout lines (*GhPH_UAV1*-Cas9) using CRISPR/Cas9 technology (Fig. 7C) and *GhPH_UAV1* overexpression lines (*GhPH_UAV1*-OE) in cotton. Three knockout lines were identified through Sanger sequencing (Fig. S3). The *GhPH_UAV1*-OE lines presented significantly elevated *GhPH_UAV1* expression in their stem tissues (Fig. 7D). Compared with that of ZM24, the height of the transgenic *GhPH_UAV1*-OE lines increased by 16.48 ​%, but that of the transgenic *GhPH_UAV1*-Cas9 cotton decreased by 21.44 ​% (Fig. 7E–F). The PH results of the *GhPH_UAV1*-OE lines and *GhPH_UAV1*-Cas9 under field growing conditions matched those under laboratory growing conditions (Fig. S4). The cotton stem tissue was cut vertically, and the length of the cells was observed. The *GhPH_UAV1*-OE lines presented significantly increased cell length, whereas the *GhPH_UAV1*-Cas9 lines presented significantly reduced cell length (Fig. 7G–H), suggesting that cell length may influence cotton PH variation. These results suggested that *GhPH_UAV1* positively regulates cotton PH.

## 4 Discussion

### 4.1 Advantages of UAV-based phenotyping platforms for the phenotypic monitoring of field crops

The objective of breeding is the selection of the most desirable phenotypes under specific conditions, so obtaining accurate phenotypic data in the field is crucial for breeding. However, manual measurement of phenotypic data is time-consuming, expensive and inaccurate, thus greatly limiting crop improvement [38,39]. UAVs are becoming more widely used in research to assess field crop phenotypes because of advantages such as rapid monitoring and high throughput [[40], [41], [42]]. A UAV equipped with RGB, LiDAR, multispectral, and hyperspectral cameras enables researchers to identify crop traits, including morphological phenotypes such as PH values, in the field throughout the life cycle [43]. Physiological phenotypes such as chlorophyll content and biomass [44,45], as well as abiotic and biotic stress indicators (leaf water potential, senescence index, and canopy temperature difference), nutrients (protein content and nitrogen concentration), and yield [46], can also be evaluated.

### 4.2 Effective monitoring of time series changes in PH in field cotton using RGB and LiDAR data

PH is the sum of aboveground internode lengths, which reflects the rate of vegetative growth in crops and is associated with plant architecture and yield performance [47]. For field crops that are experiencing rapid growth periods or rapid changes in PH under abiotic stresses such as drought, flooding, and salinity, UAV-based phenotyping provides a natural advantage for identifying and analyzing these changes [44,45,48]. To date, RGB and LiDAR data have typically been used in isolation to extract crop heights. However, the different operating principles of the three sensors and the different data analysis strategies can be validated against each other for monitoring traits in the same population. Both sensors have not been used simultaneously for monitoring time series changes in PH in cotton field populations in any previous study. A high-throughput phenotyping platform based on UAV RGB and LiDAR was developed for the first time to obtain time series PH data for 419 upland cotton accessions in the field. After these data were analyzed via linear regression and machine learning methods, the correlations between the sensor-derived data and manual measurements were found to be ranked as follows: LiDAR (R^2^ ​= ​0.934) ​> ​RGB (R^2^ ​= ​0.914). This indicated the high accuracy and feasibility of this phenotyping platform as an alternative to manual measurements at the field scale.

### 4.3 LiDAR and RGB monitoring of cotton time series PH in the field yielded good consistency in the GWAS analysis

LiDAR technology, a remote sensing method, measures the distance between the sensor and the target using an illuminating laser [49]. Owing to its high scanning frequency and wide range [50], LiDAR has great potential for plant phenotyping. The acquisition of plant height via LiDAR has been conducted in many crops, such as cotton [20], wheat [51], maize [52], and rice [53]. However, these studies were often limited to a single material or a few growth periods and did not associate the data with genotypes for further validation. To verify the effectiveness of LiDAR data obtained via this phenotyping platform for application in gene identification, a further GWAS analysis of predicted PH values combined with genotypic data identified five significant signals present across multiple time points, confirming the feasibility of LiDAR for monitoring rapid changes in cotton PH.

RGB-based crop height monitoring is widely used because of its low cost and high versatility [54], as shown in crops such as cotton [21], wheat [43], faba bean [55], peanut [56], and sorghum [57]. For cotton, only Ye et al. acquired field images of 320 cotton accessions with genotype data using a UAV equipped with a visible camera. They obtained PH predictions using linear regression based on manually measured PH data and successfully identified genes significantly associated with PH through GWAS analysis [30]. This demonstrated the important potential of RGB images acquired by UAVs for cotton PH extraction, improving the accuracy of PH predictions and their application in gene identification. We found that the accuracy was greater for RGB data obtained via machine learning than for those obtained via traditional linear regression, so different analysis strategies may also further improve the monitoring results. GWAS analysis of the RGB-based PH predictions revealed the same five significant signals across multiple time points as the results based on LiDAR data, further supporting the feasibility of RGB for monitoring rapid changes in cotton PH.

### 4.4 The GhPH_UAV gene plays a role in exploring the genetic basis of cotton PH

PH is a complex trait influenced by many factors. The identification of height-related genes is typically performed via two strategies: GWAS analysis of natural populations with relatively mild variation and manually measured phenotypic data [30,37,58] or the acquisition of data from extreme phenotypic mutants to elucidate the mechanisms governing PH [6,59]. High-throughput PH measurements via UAVs and correlations with genotype data to elucidate molecular mechanisms are currently popular research topics. Identifying PH-related genes will provide more options for modern breeding. We combined these two strategies and identified 34 genes via GWAS analysis of 419 multiperiod cotton PH datasets acquired by multiple UAV sensors with genotype data, two of which have been demonstrated to regulate cotton PH, namely, *GhUBP15* [30] and *GhPH1* [37]. Ye et al. identified the *GhUBP15* gene by acquiring field cotton PH data from only UAV RGB images and analyzing them by linear regression. The multisensor and multiple analysis method fusion strategy was used to obtain a comprehensive set of genes regulating plant height. Tian et al. identified the *GhPH1* gene by acquiring field cotton PH data using manual measurement, indicating that the phenotyping platform constructed in this study is highly accurate and feasible as an alternative to manual measurements at the field scale. We further identified significant differences in the expression of the *GhPH_UAV1* gene, which may be related to PH, in the stems of the *G. hirsutum* cultivar ZM24 compared with the *pag1* mutant. Our knockout and overexpression experiments confirmed this hypothesis, confirming the efficiency and feasibility of the strategy used in this study.

## 5 Conclusion

In this study, a high-throughput phenotyping platform based on UAV RGB and LiDAR, which is more flexible and efficient for deployment of phenotyping in the field than manual measurements or indoor phenotyping platforms, was developed, and its timeliness ensures the reliability of multiperiod monitoring of phenotypic traits in large populations. The combined use of multiple sensors coupled with machine learning provides more accurate and stable data on large field phenotypes than a single sensor, and further integration with GWAS and transcriptome data has advantages in exploring important alleles that regulate PH in natural populations.

## Author contributions

L.F., J.Y. and X.W.: Methodology, software development, data collection and processing, and writing (original draft and editing). Z.L. and B.X.: experimental field design and management and writing (original draft and editing). L.L., C.G., X.A. and F.L.: Data collection and writing (review and editing). L.G., Y.Y. and Z.Y.: Methodology and writing (review and editing).

## Data availability

The source code, images captured by UAVs, data obtained from the analysis, and other datasets supporting the results presented here are available at https://github.com/Liqiangfan/419-cotton-plant-height-datasets.

## Funding

This work was supported by the National Key R&D Program of China (2022YFF1001400), the National Natural Science Foundation of China (32360509), the Natural Science Foundation of Xinjiang Uygur Autonomous Region (2024D01A150), the Key Research and Development Program of Xinjiang Autonomous Region (2023B02014), the Corps of Agricultural Science and Technology Innovation Project Special (NCG202316), Key Research and Development Program of Xinjiang (2022B02052), the National Natural Science Foundation of China (32301888), the National Key Laboratory of Cotton Bio-breeding and Integrated Utilization (CBIU2024004), and the Natural Science Foundation of Henan (232300421253).

## Declaration of competing interest

The authors declare that they have no known competing financial interests or personal relationships that could have appeared to influence the work reported in this paper.
